# Characterization of Nociceptive Behaviors Induced by Formalin in the Glabrous and Hairy Skin of Rats

**DOI:** 10.15412/J.BCN.03080105

**Published:** 2017-01

**Authors:** Elaheh Erami, Hassan Azhdari-Zarmehri, Keiji Imoto, Hidemasa Furue

**Affiliations:** 1. Department of Nursing, Faculty of Nursing and Midwifery, Torbat Heydariyeh University of Medical Sciences, Torbat Heydariyeh, Iran.; 2. Department of Medical Basic Sciences and Neuroscience Research Center, Torbat Heydariyeh University of Medical Sciences, Torbat Heydariyeh, Iran.; 3. Department of Information Physiology, National Institute for Physiological Sciences, Okazaki, Japan.; 4. School of Life Science, University for Advanced studies (SOKENDAI), Okazaki, Japan.

**Keywords:** Formalin test, Hairy skin, Glabrous skin, Tonic pain, Nociception

## Abstract

**Introduction:**

Glabrous skin and hairy skin are innervated by different types of noxious fibers. However, the different nociceptive behaviors induced by formalin, a commonly used model of acute inflammatory pain, have not yet been systematically examined in the glabrous and hairy skin.

**Methods:**

In this study, we compared nociceptive behaviors induced by formalin injections (2%, 4%, and 8%) into either glabrous skin (plantar surface) of the hind paw or hairy skin of the hind limb in adult rats.

**Results:**

A typical biphasic nociceptive response was seen after formalin injection into the plantar surface of the hind paw. A brief interphase separates the first and second phases where nociceptive behaviors were barely spotted. However, following subcutaneous injection into the hairy skin nociceptive behaviors were only seen after 10 minutes of formalin injection, which correlates in time with the second phase of the formalin response. First phase nociceptive behaviors were never seen with hairy skin injection, even following multiple injections of formalin.

**Conclusion:**

These data suggest that nociceptive behaviors and spinal responses induced by formalin injections to glabrous and hairy skin areas are different, and that the first and second phases may be mediated through different noxious afferent fibers.

## 1. Introduction

The formalin test as an acute inflammatory pain model is a commonly used (Sofiabadi et al., 2014; Azhdari-Zarmehri, Semnanian, & Fathollahi, 2014; Azhdari-Zarmehri, Esmaeili, Sofiabadi, & Haghdoost-Yazdi, 2013; Heidari-Oranjaghi, Azhdari-Zarmehri, Erami, & Haghparast, 2012; Erami, Azhdari-Zarmehri, Ghasemi-Dashkhasan, Esmaeili, & Semnanian, 2012). When compared to other nociceptive stimuli (e.g. electrical, thermal, or mechanical stimulation), the characteristics of the formalin test (i.e. a progressive pain response, which has a relatively long duration and is inescapable) are thought to most closely mimic clinical pain (Clavelou, Dallel, Orliguet, Woda, & Robisson, 1995; Coderre, & Melzack, 1992). It is well established that localised, subcutaneous (SC) injection of diluted formalin (1%–10%) to glabrous areas of the hind paw generates behavioral nociceptive responses that are characterized by three phases (Clavelou et al., 1995; Coderre, & Melzack, 1992).

The early phase of activity (0–7 min) begins immediately following injection and reflects a direct activation of peripheral nociceptors. Following this period, the interphase begins, which is characterized by attenuation of nociceptive behaviors. The second phase (15–90 min) begins approximately 15 minutes after the injection and reflects ongoing peripheral activity and central sensitization (Coderre & Melzack, 1992; Coderre, Vaccarino & Melzack,1990; Dubuisson & Dennis, 1978; Erami, Azhdari-Zarmehri, Ghasemi-Dashkhasan, Esmaeili & Semnanian, 2012; Gheibi, Saroukhani & Azhdari-Zarmehri, 2013). Furthermore, biphasic painful behaviors (Clavelou et al., 1995; Coderre, & Melzack, 1992) as well as electrophysiological responses from dorsal horn neurons of the spinal cord, can be recorded for longer than one hour after formalin injection (Heidari-Oranjaghi, Azhdari-Zarmehri, Erami & Haghparast, 2012; Hunskaar, Berge & Hole, 1986).

Many studies have used the formalin test as an acute and tonic inflammatory pain model, including inflammatory and chronic pain states (Clavelou et al., 1995; Coderre, & Melzack, 1992). The glabrous and hairy skin are innervated by different types of nociceptors (Hunskaar & Hole, 1987). However, the different nociceptive behaviors induced by formalin injection into the glabrous vs hairy skin are not well investigated. Examining the differences in these formalin-induced nociceptive behaviours may help us to understand the mechanisms underlying the nociceptive responses that occur in different phases of the formalin test. Therefore, we assessed whether the quality of formalin-induced nociceptive behaviors differs for hairy and glabrous skin areas.

## 2. Methods

Experiments were performed on male rats (Sprague-Dawley; 170–250g, n=48, rats housed 3 per cage). All activities were confirmed by the guidelines for Animal Research Committee of National Institute for Physiological Sciences (NIPS), Okazaki, Japan. We tried to decrease the number of animals using for the studies. Animals were group-housed under a standard 12 h light/dark cycle at temperature controlled room and have with ad libitum access to food and water.

### 2.1. Formalin test

Before the inception of the experiment, rats were moved to testing lab for at least 60 minutes and put in the formalin testing boxes for habituation (H:30×W:20×L:25 cm) for at least 30 minutes. A mirror was placed underneath at a 45° angle to allow clear view of the paws. Formalin (50 μL; SC; 2%, 4%, or 8%) was injected into either the glabrous or the hairy skin of the hind limb using a 30-gauge needle inserted under the skin and advanced approximately 5–7 mm at an angle of 15–200. The formalin-induced nociceptive behaviours wree recorded using a digital video camera (DV, Sony, Japan) for 90 min to off-line analyze pain-like behaviors. Experimenters left the testing room during the recording period. These behaviours were scored as follows: 0, formalin injected paw was normal as weight bearing; 1, the formalin injected one has little weight placed on it; 2, the formalin injected one was any weight placed on it as elevated; and 3, the injected formalin paw is bitten or licked. Recording of nociceptive behaviors started after formalin injection (0 min) and was continued for 90 min. Formalin-induced nociceptive behaviours were calculated and evaluated separately during the phase 1 (1–7 min), inter-phase (8–14 min), the phase 2A (15–60 min) and the phase 2B (61–90 min) (Abbott, et al., 1995; Azhdari-Zarmehri, et al., 2011).

### 2.2. Drugs

Three different concentrations of formalin (2%, 4%, and 8%) were prepared by diluting a saturated aqueous solution of formaldehyde (37%) (Wako, Japan) with sterile physiological saline solution.

### 2.3. Data analysis

The study data (are presented as mean±standard error of the mean [SEM]) were analyzed by 1-way analysis of variance (ANOVA) followed by Tukey and t test between groups. P<0.05 was defined for statistical significance level (Abbott, et al.,1995; Azhdari-Zarmehri, et al., 2011).

## 3. Results

**Figure 1:**
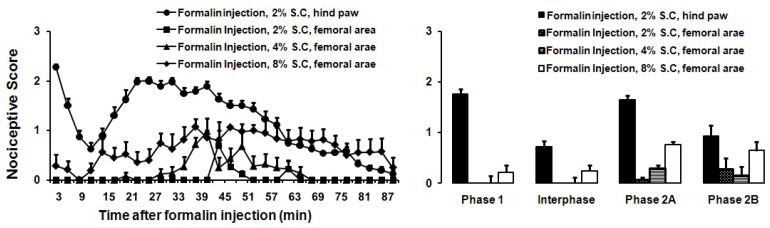
Effects of subcutaneous formalin injection (2, 4 and 8%, 50 μl) into the hairy skin of the right hind leg. A) The score of formalin induced nociceptive behaviours was measured every 3 minutes for 90 minutes (n=8). B) The columns indicated nociceptive behavioral score following formalin injection for the phase 1, inter-phase, phase 2A and phase 2B and used for following figures..

Consistent with previous studies, subcutaneous formalin administration (2%) induced nociceptive behaviors in acute (phase 1) and chronic lasting time (more than 60 min). Rats showed pain-like behaviors for the first 0–7 min followed by an interphase (8–14 min), in which nociceptive behaviors (i.e. elevation, biting, or licking of injected paw) were attenuated or stopped. The second phase began approximately 15 minutes after the formalin injection and lasted until 90 minutes (Figure 1).

**Figure 2:**
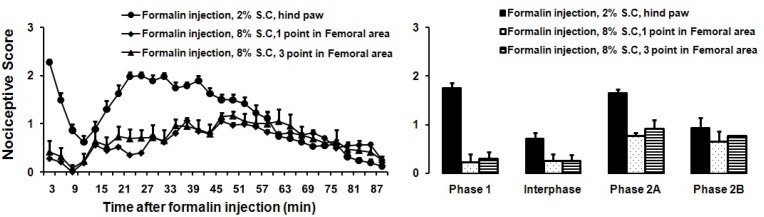
Effects of subcutaneous formalin injection (2, 4 and 8%, 50 μl) into the hairy skin of the right hind leg. A) The score of formalin induced nociceptive behaviours was measured every 3 minutes for 90 minutes (n=8). B) The columns indicated nociceptive behavioral score.

To assess whether the quality of formalin induced nociceptive behaviors differs for hairy vs. glabrous skins areas, we injected formalin subcutaneously into the hairy skin of the hind limb. Subcutaneous injection of two concentrations of formalin (2%, 4%) into the hairy skin induced nociceptive behaviors only in the second phase. Single and repeated SC injections of formalin (8%) into the hairy skin did not increase the number of nociceptive behaviors seen in the first phase (Figure 1 and 2). No pain-like behaviors were observed after control injection of saline into the hairy skin (SC) or underlying muscle (IM).

## 4. Discussion

Studies in a variety of species have revealed differences in the types of nociceptors that innervate glabrous and hairy skin. For example, recordings from primary afferent nerves in monkeys indicate that different mechanosensitive and heat nociceptors innervate hairy and glabrous skin (Hunskaar & Hole, 1987). The density, depth, and location of nociceptors are also known to differ (Kakuda, 1992; Lynn & Shakhanbeh, 1988; Malmberg & Yaksh, 1992). However, differences in the types or density of nociceptors and primary afferents that innervate hairy and glabrous skin in the rat have not been well studied. Considering many electrophysiological studies use stimulation of the hind limb hairy skin, it is important to identify whether these regions respond normally to conventional nociceptive tests, including the formalin test.

The formalin model test has some advantages in pain experiment used in our studies; formalin injection was used as adequate stimulus and continuously induced nociceptive behaviors rather than transient ones in unrestrained animals. Clinical analgesic drugs produced antinociceptive effect in this model and it is representative of many clinical pain states. Importantly, the formalin test as chemical nociceptive stimulus produces two different and separate phases, which may manifest different types of pain modulation (Mense & Prabhakar, 1986; Porro & Cavazzuti, 1993).

In the first descriptions of the formalin test, as well as in many subsequent studies, the either forepaw or the hind paw was used for the subcutaneous formalin administration. The hairy dorsal surface was most commonly used for formalin injection (Clavelou, Dallel, Orliaguet, Woda & Raboisson, 1995; Roveroni, Parada, Cecılia, Veiga & Tambeli, 2001), however, subcutaneous plantar injections have also been used. Consistent with previous studies, subcutaneous formalin administration into the right hind paw (the plantar surface) induced two phases of nociceptive responses. The short first phase lasted less than 10 min and was followed by a second phase which may reflect ongoing peripheral activity.

However, subcutaneous injection of formalin (2%, 4%, and 8%) into the hairy skin of the right hind limb did not induce nociceptive behaviors until 10 minutes after of formalin injection, correlating in time with the second phase of the formalin injection in the glabrous skin. Similarly, repeated SC injections of formalin into the hairy skin did not induce any nociceptive behaviors in the early first phase.

This result suggests that the second phase of nociceptive behavior can actually be induced in the absence of the first phase. Many analgesic substances inhibit only the second phase of the formalin response, including anti-inflammatory drugs (Sarookhani, Ghasemi-Dash-khasan, Heidari-Oranjaghi, Azhdari-Zarmehri, Erami, & Hosseini, 2014), N-methyl-D-aspartate antagonists (Sessle & Hu, 1991; Shamsizadeh, Soliemani & Mohammad-Zadeh, 2014), and morphine (Coderre & Melzack, 1992; Shrestha, Gracias, Mujenda, Khodorova, Vasko & Strichartz, 2009), again indicating that the second phase is driven by local inflammatory mediators in the periphery.

Interestingly, higher concentrations of formalin (8%) did induce a short first phase, which led to a longer second phase when compared to 2% and 4% formalin. This indicates that there is an activity-dependent enhancement of the second phase, which may be due to sensitization of neurons in the central nervous system.

Although the formalin-induced nociceptive is a reliable pain model, but we cannot assess the mechanisms of nociception which is participated in hairy skin area. Considering that pain sensations may differ for deep vs. cutaneous tissues, it is possible that the choice of formalin injection site has some impact on the intensity and pattern of the response. Unlike subcutaneous injection, deep (intramuscular) injection of formalin into the hairy area did induce a typical biphasic nociceptive response, showing a brief interphase (Erami & Azhdari-Zarmehri, 2016). Control injections of saline into hairy areas (SC or IM) did not lead to any pain-like behaviors and is consistent with other studies reported in the paw (Sofiabadi, Azhdari-Zarmehri, Naderi, Ghalandari-Shamami, Sonboli & Haghparast, 2014) and in the upper lip formalin test (Tjølsen, Berge, Hunskaar, Rosland & Hole, 1992).

A difference in nociceptive behaviors for glabrous vs hairy skin has been reported for other algogenic substances, also for cold-induced pain and prickle. For example, subcutaneous injection of endothelin-1 into the glabrous skin of the rat hind paw is known to produce acute nocifensive behavioral responses, such as hind paw flinching as well as mechanical and thermal sensitization (Treede, Meyer, Raja, & Campbell, 1995). Although subcutaneous injection of endothelin-1 into hairy skin area caused a local, transient analgesia to punctate mechanical stimulation, it was concentration-dependent (Treede, et al; 1995).

Previous studies have suggested that phase 2 of the formalin test results from peripheral inflammatory mediators and central sensitization was induced during the first phase (Hunskaar, et al; 1986; Turnbull & Rasmusson, 1986; Wheeler-Aceto, & Cowan; 1993). Based on some documents, pain disturbances occur more frequently in deep tissues (Woolf, 1983; Yaksh, Ozaki, McCumber, Rathbun, Svensson, Malkmus, & Yaksh, 2001) and behavioral changes seen with this deep model are rubbing, flinching, and head turning (Erami & Azhdari-Zarmehri, 2016). Also some documents report that pain disturbances occur in the deep tissues more than cutaneous ones (Yoon, Bae, Choi, Jeong, Chung, Yoo, et al., 2005).

It is therefore possible that changes in the central nervous system due to the afferent barrage during the early first phase, in addition to peripheral factors such as the density, depth, or location of nociceptors in hairy versus glabrous skin, may contribute to the differences seen in formalin induced nociceptive behaviors reported in the current study and elsewhere (Kakuda; 1992; Lynn, & Shakhanbeh, 1988; Malmberg, & Yaksh; 1992). As these different components of the pain experience might be modulated independently. Separate evaluation of these behaviors using superficial (subcutaneous) and deep (muscular) injection protocols may be useful for assessing the antinociceptive behaviors of the analgesic drugs.

In conclusion, our results suggest that the nociceptive behaviors induced by formalin injection of glabrous skin areas differ from that evoked by hairy skin areas.

## References

[B1] Abbott F. V. Franklin K. B. J. Westbrook F. R. (1995). The formalin test: scoring properties of the first and second phases of the pain response in rats. Journal of Pain, 60 1, 91–102. doi:10.1016/0304-3959(94)00095-v 7715946

[B2] Azhdari-Zarmehri H. A. Semnanian S. Fathollahi Y. Erami E. Khakpay R. Azizi H. Rohampour K. (2011). Intra-periaqueductal gray matter microinjection of orexin-A decreases formalin-induced nociceptive behaviors in adult male rats. Journal of Pain, 12(2), 280–87. doi: 10.1016/j.jpain.2010.09.006 21145791

[B3] Azhdari-Zarmehri H. Esmaeili M. H. Sofiabadi M. Haghdoost-Yazdi H. (2013). Orexin receptor type-1 antagonist SB-334867 decreases morphine-induced antinociceptive effect in formalin test. Pharmacology Biochemistry and Behavior, 112, 64–70. doi: 10.1016/j.pbb.2013.09.018. 24125787

[B4] Azhdari-Zarmehri H. Mohammad-Zadeh M. Feridoni M. Nazeri M. (2014). Termination of nociceptive bahaviour at the end of phase 2 of formalin test is attributable to endogenous inhibitory mechanisms, but not by opioid receptors activation. Basic and Clinical Neuroscience, 5 1, 48–57. PMCID: 25436084 PMC4202598

[B5] Azhdari-Zarmehri H. Semnanian S. Fathollahi Y. (2014). Orexin-A microinjection into the rostral ventromedial medulla causes antinociception on formalin test. Pharmacology Biochemistry and Behavior, 122, 286–90. doi: 10.1016/j.pbb.2014.03.017. 24685412

[B6] Clavelou P. Dallel R. Orliaguet T. Woda A. Raboisson P. (1995). The orofacial formalin test in rats: effects of different formalin concentrations. Pain, 62(3), 295–301. doi: 10.1016/0304-3959(94)00273-h 8657429

[B7] Coderre T. J. Melzack R. (1992). The role of NMDA receptor-operated calcium channels in persistent nociception after formalin-induced tissue injury. Journal of Neuroscience, 12(9), 3671–675. 1326611 10.1523/JNEUROSCI.12-09-03671.1992 PMC6575721

[B8] Coderre T. J. Vaccarino A. L. Melzack R. (1990). Central nervous system plasticity in the tonic pain response to subcutaneous formalin injection. Brain Research, 535(1), 155–58. doi: 10.1016/0006-8993(90)91835-5 2292020

[B9] Dubuisson D. Dennis S. G. (1978). The formalin test: a quantitative study of the analgesic effects of morphine, meperi-dine, and brain stem stimulation in rats and cats. Pain, 4(2), 161–74. doi: 10.1016/0304-3959(77)90130-0 564014

[B10] Erami E. Azhdari-Zarmehri H. Ghasemi-Dashkhasan E. Esmaeili M. H. Semnanian S. (2012). Intra-paragigantocellularis lateralis injection of orexin-A has an antinociceptive effect on hot plate and formalin tests in rat. Brain Research, 1478, 16–23. doi: 10.1016/j.brainres.2012.08.013. 22906776

[B11] Erami E. Azhdari-Zarmehri H. (2016). A rat muscle pain model based on intramuscular formalin injection. Caspian Journal of Neurological Sciences, 2(5), 22–28.

[B12] Gheibi N. Saroukhani M. Azhdari-Zarmehri H. (2013). The effect of food deprivation on nociception in formalin test and plasma levels of noradrenaline and corticosterone in rats. Basic and Clinical Neuroscience, 4 4, 341–47. PMCID: 25337367 PMC4202578

[B13] Heidari-Oranjaghi N. Azhdari-Zarmehri H. Erami E. Haghparast A. (2012). Antagonism of orexin-1 receptors attenuates swim-and restraint stress-induced antinociceptive behaviors in formalin test. Pharmacology Biochemistry and Behavior, 103(2), 299–307. doi: 10.1016/j.pbb.2012.08.007 22922083

[B14] Hunskaar S. Berge O. G. Hole K. (1986). Dissociation between antinociceptive and anti-inflammatory effects of acetylsalicylic acid and indomethacin in the formalin test. Pain, 25(1), 125–32. doi: 10.1016/0304-3959(86)90014-x 3714284

[B15] Hunskaar S. Hole K. (1987). The formalin test in mice: dissociation between inflammatory and non-inflammatory pain. Pain, 30(1), 103–14. doi: 10.1016/0304-3959(87)90088-1 3614974

[B16] Kakuda N. (1992). Conduction velocity of low-threshold mechanoreceptive afferent fibers in the glabrous and hairy skin of human hands measured with microneurography and spike-triggered averaging. Neuroscience Research, 15(3), 179–88. doi: 10.1016/0168-0102(92)90003-u 1336831

[B17] Lynn B. Shakhanbeh J. (1988). Properties of Aδ high threshold mechanoreceptors in the rat hairy and glabrous skin and their response to heat. Neuroscience Letters, 85(1), 71–76. doi: 10.1016/0304-3940(88)90431-4 3362415

[B18] Malmberg A. B. Yaksh T. L. (1992). Antinociceptive actions of spinal nonsteroidal anti-inflammatory agents on the formalin test in the rat. Journal of Pharmacology and Experimental Therapeutics, 263(1), 136–46. doi: 10.1016/0304-3940(94)90181-3 1403779

[B19] Mense S. Prabhakar N. R. (1986). Spinal termination of nociceptive afferent fibres from deep tissues in the cat. Neuroscience Letters, 66(2), 169–74. doi: 10.1016/0304-3940(86)90185-0 3725183

[B20] Porro C. A. Cavazzuti M. (1993). Spatial and temporal aspects of spinal cord and brainstem activation in the formalin pain model. Progress in Neurobiology, 41(5), 565–607. doi: 10.1016/0301-0082(93)90044-s 8284437

[B21] Roveroni R. C. Parada C. A. Cecılia M. Veiga F. A. Tambeli C. H. (2001). Development of a behavioral model of TMJ pain in rats: the TMJ formalin test. Pain, 94(2), 185–91. doi: 10.1016/s0304-3959(01)00357-8 11690732

[B22] Sarookhani M. R. Ghasemi-Dashkhasan E. Heidari-Oranjaghi N. Azhdari-Zarmehri H. Erami E. Hosseini S. S. (2014). Effect of Food Deprivation on Formalin-Induced Nociceptive Behaviors and Beta-Endorphin and Sex Hormones Concentration in Rats. Iranian Biomedical Journal, 18(2), 107–13. doi: 10.1016/j.pbb.2012.08.007 24518552 PMC3933920

[B23] Sessle B. J. Hu J. W. (1991). Mechanisms of pain arising from articular tissues. Canadian Journal of Physiology and Pharmacology, 69(5), 617–26. doi: 10.1139/y91-092 1863912

[B24] Shamsizadeh A. Soliemani N. Mohammad-Zadeh M. (2014). Permanent lesion in rostral ventromedial medulla potentiates swim stress-induced analgesia in formalin test. Iranian Journal of Basic Medical Sciences, 17 3, 209–15. PMCID: 24847424 PMC4016692

[B25] Shrestha S. Gracias N. G. Mujenda F. Khodorova A. Vasko M. R. Strichartz G. R. (2009). Local antinociception induced by endothelin-1 in the hairy skin of the rat’s back. Journal of Pain, 10(7), 702–14. doi: 10.1016/j.jpain.2008.12.005 19559389 PMC2720057

[B26] Sofiabadi M. Azhdari-Zarmehri H. Naderi F. Ghalandari-Shamami M. Sonboli A. Haghparast A. (2014). Effects of Hydroalcoholic Extract of Tanacetum Sonbolii (Asteraceae) on Pain-related Behaviors during Formalin Test in Mice. Basic and Clinical Neuroscience, 5 2, 162–68. PMCID: 25337375 PMC4202584

[B27] Tjølsen A. Berge O. G. Hunskaar S. Rosland J. H. Hole K. (1992). The formalin test: An evaluation of the method. Pain, 51(1), 5–17. doi: 10.1016/0304-3959(92)90003-t 1454405

[B28] Treede R. D. Meyer R. A. Raja S. N. Campbell J. N. (1995). Evidence for two different heat transduction mechanisms in nociceptive primary afferents innervating monkey skin. Journal of Physiology, 483(3), 747–58. doi: 10.1113/jphysiol.1995.sp020619 7776255 PMC1157815

[B29] Turnbull B. G. Rasmusson D. D. (1986). Sensory innervation of the raccoon forepaw: 1. Receptor types in glabrous and hairy skin and deep tissue. Somatosensory Research, 4(1), 43–62. doi: 10.3109/07367228609144597 3797914

[B30] Wheeler-Aceto H. Cowan A. (1993). Naloxone causes apparent antinociception and pronociception simultaneously in the rat paw formalin test. European Journal of Pharmacology, 236(2), 193–99. doi: 10.1016/0014-2999(93)90589-a 8319750

[B31] Woolf C. J. (1983). Evidence for a central component of post-injury pain hypersensitivity. Nature, 306(5944), 686–88. doi: 10.1038/306686a0 6656869

[B32] Yaksh T. L. Ozaki G. McCumber D. Rathbun M. Svensson C. Malkmus S. Yaksh M. C. (2001). An automated flinch detecting system for use in the formalin nociceptive bioassay. Journal of Applied Physiology, 90(6), 2386–402. doi: 10.1097/00000542-199502000-00012 11356806

[B33] Yoon M. H. Bae H. B. Choi J. I. Jeong S. W. Chung S. S. Yoo K. Y. (2005). Evaluation of interaction between intrathecal adenosine and MK801 or NBQX in a rat formalin pain model. Pharmacology, 75(3), 157–64. doi: 10.1159/000088345 16166819

